# Plant Extracts as Functional Food Ingredients

**DOI:** 10.3390/foods14030374

**Published:** 2025-01-23

**Authors:** Jaroslawa Rutkowska, Antonella Pasqualone

**Affiliations:** 1 Institute of Human Nutrition Sciences, Faculty of Human Nutrition, Warsaw University of Life Sciences (WULS-SGGW), Nowoursynowska st.159c, 02-776 Warsaw, Poland; 2 Department of Soil, Plant and Food Science (DISSPA), University of Bari ‘Aldo Moro’, Via Amendola 165/a, 70126 Bari, Italy; antonella.pasqualone@uniba.it

## 1. Introduction

Plant extracts are a source of phytochemicals with multiple biological activities, so they have always been the basis for the composition of pharmaceuticals, supplements, and functional foods. In fact, the recognition of the relationship between food and health has led to growing demand for functional foods that provide additional physiological benefits beyond their basic nutrition [1]. The key aims of this research area are the identification of new plant sources, the development of effective extraction methods to recover plants’ bioactive compounds, and the study of new food applications for the obtained extracts. These steps are complemented by appropriate qualitative–quantitative analyses of both the extracts and the enriched foods to assess their biological properties (in vitro and in vivo) while evaluating the effect of the extracts on the physicochemical and sensory characteristics of the foods being functionalized.

The bioactive compounds typically present in plant extracts can be highly diverse and can be hydrophilic or lipophilic in nature; they include polyphenols, carotenoids, chlorophylls, tocopherols and tocotrienols, phytoestrogens, phytosterols, polyunsaturated fatty acids, peptides, and polysaccharides. The extraction of these compounds is conditioned by their chemical nature and can be modified by various factors (pH, temperature, pressure) to increase yield and, if possible, improve environmental safety. Several technologies have recently been developed for more environmentally friendly extraction of functional compounds from plant sources, such as ultrasound-assisted extraction (UAE), supercritical (SC) fluid extraction, microwave-assisted extraction (MAE), pulsed electric fields (PEF), and pressurized liquid extraction (PLE) [2,3,4,5], although their full industrial scalability is still under study. Also, the use of natural deep eutectic solvents (NADES) has been proposed as a green option compared to most organic solvents commonly used in extraction processes (e.g., methanol, acetone, hexane) [4,5].

Thermomechanical stress associated with the processing steps in food preparation can degrade bioactive compounds, necessitating the adjustment of processing parameters by choosing milder conditions where possible [6]. Once bioactive compounds have been added to food, the digestion process can affect their bioavailability and beneficial effects [7]. Effective encapsulation methodologies are thus needed to improve the stability of bioactive compounds and preserve their characteristics until they are delivered to the biological target [8].

Besides having a positive effect on health, functional foods may also possess improved technological features, such as longer storability, compared to conventional foods in the same category. So, another benefit of the addition of plant extracts is improved shelf-life, generally observed when the added compounds possess antioxidant properties [9]. However, we must carefully consider the effect of plant extracts on the physicochemical and especially sensory characteristics of foods. Negative sensory effects and color alterations may result from supplementation, which could be mitigated by balancing the amount of extract added or through encapsulation, which is useful in reducing undesirable flavors or tastes [10,11]. Future research addressing this problem is essential to significantly improve consumers’ acceptance of these foods.

This Special Issue presents articles that discuss all of the abovementioned research directions, from optimizing extraction methods to evaluating the effects of extracts as new ingredients (including assessing the impact of digestion), and thus represents a valuable resource for researchers and food industry professionals.

## 2. Overview of the Contributions

Thirty-six articles were submitted to this Special Issue. Following a rigorous review process, 16 articles were published, including 14 research articles, 1 review, and 1 communication. The articles published in this Special Issue address the most recent advances in this field.

Plants are a valuable source of polysaccharides and have received much attention from researchers due to their strong biological activity, low cytotoxicity, safety, and beneficial roles in the modulation of gut microbiota composition. In this Special Issue, three papers present new knowledge about plant polysaccharides: Phomkaivon et al. [contribution 1], Wu et al. [contribution 6], and Fang et al. [contribution 16].

Green macroalgae *Ulva rigida* C. are rich in biologically active phytochemicals and possess high levels of polysaccharides (about 18% of their dry weight). Phomkaivon et al. [contribution 1] conducted a study on water extraction to obtain the maximum level of polysaccharides from oven-dried *Ulva rigida* (green macroalgae). The extraction factors analyzed included the biomass–solvent ratio, extraction temperature, extraction time, and extraction cycle. The results indicated that higher extraction temperatures led to a significant increase in polysaccharide yields. Polysaccharide extracts obtained from *Ulva rigida* exhibited antioxidative potential, probably because of the substantial presence of phenolics and flavonoids. The supplementation of polysaccharides extracted from *Ulva rigida* promoted *Lactobacillus rhamnosus* growth during cultivation. These findings demonstrated that polysaccharide extracts from *Ulva rigida* have the potential to serve as functional ingredients future food products, with numerous applications in the nutraceutical and pharmaceutical industries.

Tartary buckwheat green leaves, underappreciated by-products in the buckwheat industry, are rich in pectic polysaccharides. Wu et al. [contribution 6] aimed to optimize the extraction conditions of pectic polysaccharides from Tartary buckwheat green leaves using deep-eutectic-solvent-based techniques. The efficiency and selectiveness of these methods were compared with those of conventional hot water. The results revealed that both deep-eutectic-solvent extraction (DESE) and high-pressure-assisted deep-eutectic-solvent extraction (HPDEE) not only improved the extraction efficiency of pectic polysaccharides from Tartary buckwheat green leaves (TBP), but also regulated their structural properties and beneficial effects. In addition to high extraction efficiency, both methods enabled the extraction of valuable rhamnogalacturonan-I (RG-I) pectic fractions. High antioxidative potential and anti-glycosylation properties were noted in TBP extracts obtained using DESE and HPDEE. These findings demonstrated that developed deep-eutectic-solvent-based extraction methods, especially the HPDEE method, are promising techniques for obtaining selective RG-I from TBP.

Fang et al. [contribution 16] comprehensively reviewed the most recent literature about Konjac glucomannan (KGM), a major polysaccharide from the corm of *Amorphophallus* konjac plants that grow in Southeast Asia and Africa. KGM, a hydrocolloid dietary fiber, has attracted increasing attention as a bioactive polysaccharide due to its functional and nutraceutical features. Because of its water absorptivity, stability, film-forming, thickening, and emulsifying properties, KGM is used as a food additive. In the review by Fang et al. [contribution 16], the chemical structure, physicochemical properties, extraction, and purification process of KGM were discussed. On an industrial scale, KGM is obtained via dry-processing, which includes the washing, peeling, slicing, fixing, drying, grinding, and screening of KGM. In the wet-processing of KGM, a liquid medium is used that dissolves the KGM and removes soluble impurities. Using some solvents, ethanol prevents agglomeration. The damp method has many advantages, but is more expensive than dry methods. Regarding KGM’s structure and properties, its high molecular weight and ability to form high viscosity and adsorb water were underlined. Its physical and chemical properties make KGM an excellent preservative and fat substitute to maintain the proper texture in foods for diabetic patients. Using KGM is beneficial in food products with attributes important for diabetes, including a low-glycemic index, reduced fat, and the ability to maintain weight loss and fullness. The review by Fang et al. [contribution 16] also discusses the biomedical function of KGM in type 2 diabetes mellitus, specifically regulating blood lipids, glucose homeostasis, oxidative stress and inflammation, and the gut microflora. KGM was shown to have the most significant potential for lowering LDL and total cholesterol, promoting weight loss, and aiding in diabetic control. The administration of KGM to diabetic rats had an anti-diabetic effect by increasing gene up-regulation and insulin pathway expression. This resulted in normalized insulin secretion and lowered blood sugar levels. KGM positively regulates oxidative stress and inflammation, indicating that it may effectively prevent or treat diabetes. In conclusion, Fang et al. [contribution 16] indicated that KGM can be used as a food additive to improve food products’ taste, flavor, and appearance. However, the authors indicated that high doses of KGM may cause minor side effects such as hiccups, bloating, and diarrhea, and there is a risk of choking because of its high water absorption capacity.

In recent years, there has been significant interest in utilizing by-products and waste as sources of valuable functional ingredients [contributions 3 and 4]. In the study by Meng et al. [contribution 3], walnut meal was used as a raw material to prepare polypeptide, and its angiotensin-converting enzyme (ACE)-inhibitory activity was investigated. The fraction of ACE exhibited strong antioxidant capacity and total reducing power in vitro. The ACE inhibition retention rate was more than 90% after in vitro simulated gastrointestinal digestion, which provided a reference for the further development of ACE-inhibiting peptides in walnuts. The main finding of Meng et al.’s study provides a reference for applying low-molecular-weight walnut peptide as an antioxidant and ACE inhibitor. Glišic et al. [contribution 4] utilized post-harvest sunflower and maize stalk residues to prepare ethanolic extract, which can be used as a functional ingredient in pork liver pâtés. A substantial level of phenolic compounds was noted in the maize stalk residue extract and sunflower stalk ethanolic extract. When incorporated into pork liver pâtés, these extracts exhibited a noticeable antimicrobial effect. The crop ethanolic extracts increased the n-6 and total PUFA contents in the pâtés and improved the PUFA/SFA ratio. However, they proved ineffective in preventing lipid oxidation. Adding crop residue extracts influenced the instrumental color and intensified the flavor and aroma of the pork liver pâtés, resulting in lower product acceptability. The results of Glišic’ et al. [contribution 4] indicated the potential of these extracts obtained from agricultural residues as a source of natural preservatives for meat product applications. These findings could be valuable in developing functional pork liver pâtés without added synthetic preservatives.

Flaxseed and rapeseed are emerging as key sources of phytochemicals in the functional food arena. In addition to being one of the richest sources of PUFAs, especially α-linolenic acid, flaxseed, and rapeseed, they are considered an essential source of high-quality protein. Jarošová et al. [contribution 2] evaluated the protein profiles of flaxseed products with different protein contents (seed cake, flour, and protein concentrate). The proteomic profile of flaxseed products included 2560 protein groups; among them, 33 had a relative abundance within the protein pool of 69–95%. Alkaline solubilization increased the relative abundance of 11S globulin-type proteins in the resulting protein concentrate, which have high potential for food applications due to their properties and amino acid composition. Selecting a suitable cultivar could be an important factor in obtaining a protein concentrate with the highest abundance of 11S globulins.

Amarowicz et al.’s study [contribution 7] thoroughly examined rapeseed’s antioxidative potential and phenolic compounds. Rapeseed was derived from three varieties (Castilla, California, and Nelson F1) and was cultivated using different nitrogen and sulfur fertilization techniques. Diversifying fertilization significantly influenced the content of phenolic compounds in extracts of rapeseed. Intensive fertilization reduced the content of phenolic compounds in the Nelson F1 seeds, while economic fertilization decreased the phenolic content in the California variety. Antioxidant assessments through ABTS and FRAP assays revealed cultivar-specific responses. The California variety exhibited higher antioxidant activity in intensively cultivated seeds, whereas the Nelson F1 variety showed stronger antiradical activity in spare-cultivated seeds. These findings highlight the complex relationship between fertilization practices, phenolic compound accumulation, and the antioxidant activity of rapeseed.

In the human diet, fruits are a source of functional ingredients such as polyphenols (flavonoids, flavonols, and anthocyanins), phenolic acids, carotenoids, and vitamins. In this Special Issue, three papers analyzed the composition and properties of fruit extracts: Meremäe et al. [contribution 8], Dominguez-Valencia et al. [contribution 9], and Stockton and Al-Dujaili [contribution 15].

Meremäe et al. [contribution 8] studied the antibacterial effect of aqueous extract and 30% ethanolic extracts of chokeberry, blackcurrant, and rowan berries and their pomaces on the growth of *Listeria monocytogenes*, *Staphylococcus aureus*, *Escherichia coli*, and *Campylobacter jejuni*. Also, the polyphenolic profile and antioxidant activity of the extracts of the analyzed berries and their pomaces were investigated. The ethanolic extracts of chokeberry and blackcurrant berries and their pomaces were found to contain the highest levels of polyphenols and antioxidative potential compared with rowan berries and their pomace. The antibacterial activity of the extracts depended on the extraction solvent and mainly occurred for the ethanolic extracts. The ethanolic chokeberry extract had the most potent antibacterial activity against *Staphylococcus aureus*, which could be explain by it possessing the highest content of total polyphenols. Also, the antibacterial effect of ethanolic and aqueous extracts of blackcurrant and its pomace against Staphylococcus aureus and *Listeria monocytogenes* should be highlighted. This is probably due to the more diverse polyphenolic composition and higher content of anthocyanins (e.g., delphinidin rutinoside, cyaniding hexoside 2, and cyanidin rutinoside) in blackcurrant and its pomace compared with other berries and their pomaces. The findings obtained by Meremäe et al. [contribution 8] suggest that chokeberry and blackcurrant and their pomaces are a good source of polyphenols with antioxidative properties, and they also exhibit antibacterial activity against some foodborne pathogenic bacteria.

Dominguez-Valencia et al. [Contribution 9] characterized the hydrophilic compounds extracted from elderberries and the main lipophilic bioactive compounds retained in their solid residues. Also, their study investigated the effect of encapsulation on the concentration of hydrophilic bioactive compounds and their antioxidant capacity. The polyphenol profile of hydrophilic elderberry extract was rich with cyanidin-derived anthocyanins, characterized by high amounts of cyanidin-3-O-glucoside and cyanidin-3-O-sambubioside and non-anthocyanins compounds, such as hydroxycinnamic acids (5-O-caffeoylquinic acids) and flavonols (rutin). Encapsulation led to a significant reduction in the extract’s phenolic and anthocyanin contents and antioxidant capacity, but it also made the extract more stable. Solid residues of elderberry extracts were rich in macronutrient lipids (about 17 g/100 g) and proteins (about 15.7 g/100 g). A high content of essential fatty acids and high proportions of tocopherols were noted in the lipophilic fraction of the solid residue. The critical finding of Dominguez-Valencia et al. [contribution 9] was the development of a process for obtaining a novel, stable, and versatile food ingredient (encapsulated elderberry extract). This process includes extracting and stabilizing (encapsulating) hydrophilic compounds (mainly phenolics and anthocyanins), utilizing solid residue to extract lipophilic bioactive compounds, and possibly producing protein isolates.

Polyphenols in fruits have received much attention because of their health-promoting functionalities. Stockton and Al-Dujaili [contribution 15] evaluated the effect of pomegranate extract and juice intake on satiety parameters in healthy volunteers. The experimental group included twenty-eight subjects (men and women aged 18–65 years) with a body mass index (BMI) between 18 and 34.9 kg/m^2^. Over 3 weeks, the participants consumed pomegranate capsules (210 mg punicalgin) served with pomegranate juice. After this period, the participants recorded their satiety using visual analog scales. The results of the study were promising. Participants who consumed pomegranate capsules had higher satisfaction and feelings of fullness than those in the placebo group. The authors declared that further trials with a larger number of volunteers and an assessment of the effect of the pomegranate extract and juice on satiety parameters are required.

While plants are valuable sources of bioactive phytochemicals that can be used as functional ingredients and nutraceuticals in food products, it is also essential to evaluate their medicinal properties. Kelebek et al. [contribution 5] focused on studying the effects of infusion time, temperature, and solvent conditions on the color, antioxidant capacity, total phenolic content, phenolic profile, and antimicrobial activity of Guayusa (*Ilex guayusa* Loes.) ethanolic and water extracts. The phenolic profile of the extracts included 29 compounds with a dominant share of chlorogenic acid and its derivatives. The highest phenolic content in water and ethanolic extract was obtained at 70 °C-8 h. Antimicrobial activity analysis showed that Guayusa tea extracts generally have high levels of activity against Gram-positive pathogenic bacteria. Depending on the digestion stage, the total levels of phenolic compounds in the samples prepared with both solvents increased after oral intake. These findings provide valuable insights into functional foods, with implications for the tea industry and public health.

Nunes-Silva et al. [contribution 12] conducted a comprehensive analysis of decoctions, infusions, and hydroethanolic extracts of three plants, lemon balm (*Melissa officinalis* L.), sage (*Salvia officinalis* L.), and spearmint (*Mentha spicata* L.), to assess their polyphenolic profiles, bioactive properties, and anti-inflammatory and cytotoxic properties. The extracts had a higher content of total phenolic acids than total flavonoids. Some phenolic acids, rosmarinic, salvianolic, and lithospermic acid A, were assayed in all extracts. The extracts revealed antimicrobial activity against primary foodborne pathogens. Among the studied extracts, spearmint extract showed anti-inflammatory potential. It should be noted that all analyzed extracts showed antibacterial, antifungal, and antioxidant potential. The findings of this investigation emphasize the potential value of sage, spearmint, and lemon balm extracts as natural food ingredients to prevent spoilage, provide health benefits, and replace artificial additives in food products.

Kim et al. [contribution 10] demonstrated the preventive effect of *Allium macrostemon* Bunge extract against dysfunction in adipose tissue and the liver under co-exposure to bisphenol A (BPA) and a high-fat diet using a mouse model. *Allium macrostemon* supplementation improved adipose tissue dysfunction and reduced hepatic stress levels in the high-fat-diet- and BPA-treated mice. The authors underlined that further research investigating the specific impact of *Allium macrostemon* Bunge extract is necessary to determine its suitability as a potential food component to alleviate the harmful effect of these inflammation factors.

Calonico and De La Rosa-Millan [contribution 11] analyzed the role of bioactive compounds of hot water extracts of the Mexican medicinal plants *Ludwigia octovalvis*, *Cnidoscolus aconitifolius*, and *Crotalaria longirostrata* against some digestive enzymes. All three plant extracts exhibited enzymatic inhibition. The highest antioxidative potential showed extracts obtained from Cnidoscolus aconitifolius. The study stated that flavonoids can inhibit the activity of α-amylase and α-glucosidase, pivotal enzymes involved in the breakdown of carbohydrates.

*Cuminum cyminum* L. (cumin) is a herbaceous plant from the *Apiaceae* family. Cumin seeds are rich in bioactive constituents, such as terpenes, phenols, and flavonoids, and are appreciated for their many biological functions. Ishida et al. [contribution 14] investigated the effect of the oral administration of cumin seed aqueous extract in ovalbumin-induced allergic rhinitis. The study was conducted using mice as animal models. The in vivo experiments showed that the oral administration of cumin seed aqueous extract reduced the sneezing frequency in mice with allergic rhinitis induced by ovalbumin. Their findings suggest that ingesting aqueous extract of cumin seeds improves T-cell balance and alleviates the allergy symptoms of allergic rhinitis in vivo. These findings may provide insights into the biological functions of cumin seed ingredients in functional foods and contribute to the effective utilization of cumin seed residue after oil extraction.

Pagliari et al. [contribution 13] evaluated cinnamon *Cinnamomum verum* J. Presl bark extract with respect to its antioxidant and anti-inflammatory bioactivity after simulated digestion. They analyzed the chemical profiles of the pure and digested extracts, as well as their cellular effects, in in vitro models such as Caco2 and the intestinal barrier. The results showed that the digestive process reduces the total content of polyphenols, especially tannins, while preserving other bioactive compounds, such as cinnamic acid. These findings revealed that aqueous solvents can maintain the beneficial properties of cinnamon bark. Also, their antioxidant and anti-inflammatory properties were maintained after digestion.

## 3. Conclusions

This Special Issue showcases the diverse approaches to using eco-sustainable processes to obtain plant extracts. The findings of the studies indicated that using aqueous solvents can maintain the properties of plant extracts that are beneficial for human health. The studies also underscored innovations in utilizing agro-by-products to obtain rich bioactive extracts. These studies align with the goal of developing sustainable systems, reducing waste, and promoting healthier diets and could be of interest to the producers of functional food to encourage the effective utilization of plant extracts in the development of new products.

## Data Availability

Not applicable.
